# Influence of Deep Invasive Tumor Thrombus on the Surgical Complexity and Prognosis of Patients With Non-Metastatic Renal Cell Carcinoma Combined With Venous Tumor Thrombus

**DOI:** 10.3389/fonc.2022.833780

**Published:** 2022-02-09

**Authors:** Xun Zhao, Ye Yan, Jing-han Dong, Zhuo Liu, Hong-xian Zhang, Cheng Liu, Lu-lin Ma

**Affiliations:** Department of Urology, Peking University Third Hospital, Beijing, China

**Keywords:** deep invasive tumor thrombus, non-metastatic renal cell carcinoma, venous tumor thrombus, prognosis, surgical complexity

## Abstract

**Background:**

To evaluate the impact of deep invasive tumor thrombus (DITT) on the surgical complexity and prognosis of patients with renal cell carcinoma with venous tumor thrombus.

**Methods:**

We retrospectively reviewed clinical data of 138 patients with non-metastatic renal cell carcinoma combined with venous tumor thrombus, who underwent surgical treatment in Peking University Third Hospital from January 2015 to June 2020. Patients were divided into the DITT group (84 patients) and non-invasive tumor thrombus (NITT) group (54 patients). Chi-square, *t*-test and Mann–Whitney *U* test were used for categorical and continuous variables, respectively. Kaplan–Meier plots were performed to evaluate the influence of DITT. Univariable and multivariable Cox regressions were conducted to determine independent prognostic factors and then assembled to make a nomogram to predict the survival. The performance of the nomogram was evaluated by Harrell’s consistency index (C-index) and calibration plot.

**Results:**

Deep invasive tumor thrombus significantly increased the difficulty of surgery for patients with renal cell carcinoma with venous tumor thrombus, which is mainly reflected in longer operation time (*p* < 0.001), more surgical bleeding (*p*  < 0.001), a higher proportion of perioperative blood transfusion (*p*  = 0.006), a higher proportion of open surgery (*p* = 0.001), a longer postoperative hospital stay (*p* = 0.003), and a higher proportion of postoperative complications (*p* = 0.001). DITT (hazard ratio [HR] = 2.781, *p* = 0.040) was one of the independent risk factors for worse prognosis. Multivariate analysis showed that sarcoma-like differentiation (*p* = 0.040), tumor thrombus invasion (*p* = 0.040), low hemoglobin (*p* = 0.003), and pathological type (*p* < 0.001) were independent prognostic factors. The nomogram, combining all these predictors, showed powerful prognostic ability with a C-index of 78.8% (CI: 71.2%–86.4%). The predicted risk closely matches the observed recurrence probability.

**Conclusion:**

Deep invasive tumor thrombus significantly increased the difficulty of surgeries for patients of renal cell carcinoma with venous tumor thrombus, and may lead to poor prognosis.

## Introduction

Renal cell carcinoma (RCC) is a common malignancy of the urinary system, accounting for 2% to 3% of adult malignant tumors (1). Among them, 4% to 10% of patients will develop venous tumor thrombus (VTT) (2). Radical nephrectomy and thrombectomy can effectively improve the prognosis. After complete removal of the tumor and tumor thrombus, a 5-year survival rate of more than 50% can be achieved, while the 5-year survival rate is only about 10% when the resection is incomplete (3). Deep invasive tumor thrombus (DITT), which means that the tumor thrombus has invasion to the venous wall, can significantly increase the difficulty of surgery, and even requires partial or segmental resection of the inferior vena cava (IVC) (4). Whether DITT will lead to the poor prognosis of patients with RCC and VTT is still controversial, and there are few related reports. The purpose of this article is to evaluate the impact of DITT on surgical difficulty and prognosis.

## Patients and Methods

### Patients

We retrospectively analyzed the clinical data of patients of RCC with VTT who were admitted to the Department of Urology, Peking University Third Hospital from January 2015 to June 2020. The inclusion criteria were as follows: (a) preoperative examination confirmed renal mass with VTT; (b) patients underwent radical nephrectomy and thrombectomy; and (c) postoperative pathology was RCC. The exclusion criteria were as follows: (a) preoperative examination showed distant metastasis; (b) lymph node metastasis was found in postoperative pathology; (c) postoperative pathology was T4 stage; and (d) patients with incomplete information records and patients who were lost to follow-up. At last, a total of 138 patients were included in the study. Among them, there were 107 males and 31 females. Eighty-nine tumors were on the right side and 49 tumors were on the left side. According to Mayo classification, 32 cases had level 0 VTT, 23 cases had level I VTT, 58 cases had level II VTT, 11 cases had level III VTT, and 14 cases had level IV VTT.

**Figure 1:**
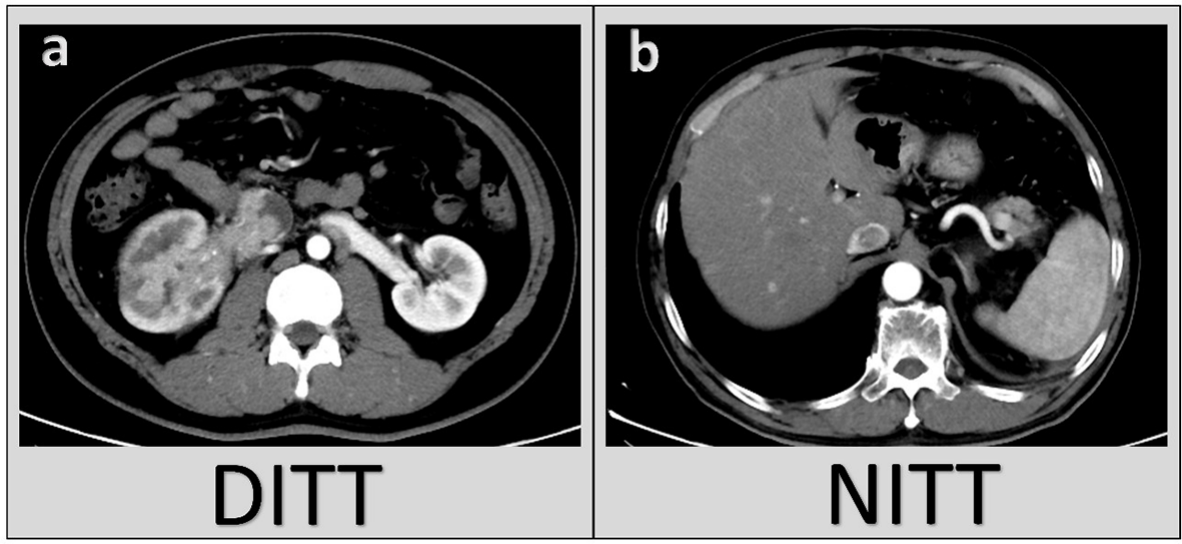
Typical CT image appearances in the Deep Invasive Tumor Thrombus (DITT) group **(A)** and the Non-Invasive Tumor Thrombus (NITT) group **(B)**.

In the study, 84 (60.9%) patients with RCC and VTT were classified to the DITT group; 54 (39.1%) cases did not have venous wall invasion, which were assigned to the non-invasive tumor thrombus (NITT) group. For renal vein tumor thrombus, the criterion for invading the venous wall is postoperative pathological result. For IVC tumor thrombus, the criterion is mainly based on intraoperative findings and partly postoperative pathological results. In this study, we also reviewed every preoperative computed tomography (CT) and/or magnetic resonance imaging (MRI) of all patients to assist in the diagnosis of DITT (**Figure 1**).

Perioperative clinical data were collected, including age, gender, laterality, body mass index (BMI), hemoglobin (Hb), neutrophil count (Neu), platelet count (Plt), albumin (Alb), serum calcium (Ca), alkaline phosphatase (ALP), preoperative serum creatinine (SCr), and Scr within 1 week after surgery, American Society of Anesthesiologists (ASA) score, lymph node metastasis status, pathological type, and Fuhrman grade. Intraoperative parameters were collected and analyzed to evaluate the impact of DITT on surgical complexity, including operation time, intraoperative bleeding, blood transfusion, postoperative complications, and postoperative hospital stay.

### Follow-Up

All patients were followed up once every 6 months until year 5, and once every year thereafter. All patients were followed up by outpatient clinic or telephone to obtain prognostic information.

### Statistical Analysis

Continuous variables with normal distribution were shown as the mean ± standard deviation and analyzed using independent sample *t*-test, while continuous variables with non-normal distribution were shown as the median (Q1, Q3) and analyzed using the Mann–Whitney *U* test. The categorical variables were summarized by percentage, and a chi-square test was performed. Univariate and multivariate Cox regression analyses were conducted to determine the important prognostic factors of overall survival (OS) by the backward stepwise method, and the factors included in the regression analysis include DITT, hemoglobin (Hb), sarcomatoid differentiation, pathological type, Fuhrman grade, IVC resection, Mayo classification, operation approach, operation time, intraoperative bleeding, postoperative hospital stay, alkaline phosphatase (ALP), neutrophil count (Neu), albumin (Alb) and clinical symptoms. Kaplan–Meier plots were performed to evaluate the influence of DITT on OS. Use SPSS version 24.0 (IBM Corporation, USA) for statistical analysis. Survival time is from the date of surgery to the date of death or the date of last follow-up.

Based on the results of multivariate analysis, a nomogram was constructed to predict postoperative 1-year, 3-year, and 5-year survival rates. The discrimination performance of the nomogram was evaluated by the consistency index (C-index). Based on 1,000 bootstrap resampling, the nomogram calibration was studied from a graphical representation of the agreement between predicted probabilities and observed results. These analyses were performed *via* R version 3.5.1 and a double-sided *p*-value of <0.05 was considered statistically significant.

## Results

The clinical and pathological data of all patients are shown in **Table 1**. The two groups had no differences in age, gender, BMI, tumor side, clinical symptoms, ASA grade, hemoglobin, neutrophils, albumin, pathological type, and nuclear grade. Compared with the NITT group, the DITT group had statistically higher Mayo classification (*p* = 0.010). In terms of the impact on surgical complexity and postoperative recovery, the DITT group had longer operation time (*p* < 0.001), more surgical blood loss (*p*  < 0.001), more surgical blood transfusion (*p*  = 0.006), more amount of plasma infusion (*p*  < 0.001), a higher proportion of open surgery rate (*p* = 0.001), a higher proportion of IVC segmental resection rate (*p*  <0.001), a longer postoperative hospital stay (*p* = 0.003), and more postoperative complications (*p* = 0.001) compared with the NITT group. Obviously, DITT increases the difficulty of nephrectomy and thrombectomy.

**Table 1:** Comparison of clinical and pathologic features between the Deep Invasive Tumor Thrombus (DITT) group and the Non-Invasive Tumor Thrombus (NITT) group.

	DITT (*N* = 84)	NITT (*N* = 54)	*p*
Mean value ± SD			
Age, years	60.2 ± 9.6	60.4 ± 11.1	0.895
BMI, kg/m ^2^	24.8 ± 3.9	24.0 ± 3.8	0.253
Tumor diameter, cm	8.1 ± 2.7	8.3 ± 3.0	0.633
Hb, g/L	122.1 ± 22.6	129.7 ± 22.5	0.055
Neu, 10^9^/L	4.7 ± 1.6	4.5 ± 2.1	0.518
Plt, 10^9^/L	241.2 ± 98.9	248.1 ± 73.2	0.659
ALP, U/L	96.0 ± 47.5	88.0 ± 31.4	0.282
Alb, g/L	38.7 ± 6.1	39.9 ± 4.8	0.228
Ca, mg/L	2.3 ± 0.2	2.3 ± 0.1	0.714
SCr, μmol/L	98.8 ± 23.3	91.3 ± 19.9	0.053
SCr after surgery, μmol/L	128.1 ± 114.6	113.7 ± 94.4	0.440
Median (Q1, Q3)			
Postoperative hospital stay, days	9.0 (7.0,13.0)	6.5 (6.0,9.0)	**0.003**
Operative time, min	338.0 (263.0,421.0)	261.0 (177.0,356.5)	**<0.001**
Surgical bleeding volume, ml	800 (280,2300)	350 (55,800)	**<0.001**
Surgical blood transfusion volume, ml	400 (0,1400)	0 (0,400)	**0.006**
Plasma transfusion volume, ml	0 (0,400)	0 (0,0)	**<0.001**
*N* (%)			
Sex			0.106
Male	69 (82.1%)	38 (70.4%)	
Female	15 (17.9%)	16 (29.6%)	
Side			0.949
Left	30 (35.7%)	19 (35.2%)	
Right	54 (64.3%)	35 (64.8%)	
ASA score			0.107
1	5 (6.0%)	3 (5.6%)	
2	64 (76.2%)	48 (88.9%)	
3	15 (17.9%)	3 (5.6%)	
Clinical symptoms			0.273
No clinical symptoms	21 (25.0%)	17 (31.5%)	
Local symptoms	43 (51.2%)	30 (55.6%)	
Systemic symptoms	20 (23.8%)	7 (13.0%)	
Surgical approach			**0.001**
Laparoscopic surgery	33 (39.3%)	37 (68.5%)	
Open surgery	51 (60.7%)	17 (31.5%)	
Mayo classification			**0.010**
0	14 (16.7%)	18 (33.3%)	
I	10 (11.9%)	13 (24.1%)	
II	40 (47.6%)	18 (33.3%)	
III	10 (11.9%)	1 (1.9%)	
IV	10 (11.9%)	4 (7.4%)	
IVC resection			**<0.001**
No	56 (66.7%)	54 (100.0%)	
Yes	28 (33.3%)	0 (0.0%)	
Pathology type			0.218
Clear cell carcinoma	70 (83.3%)	49 (90.7%)	
Non Clear cell carcinoma	14 (16.7%)	5 (9.3%)	
Fuhrman Grade			0.147
I-II	33 (39.3%)	28 (51.9%)	
III-IV	51 (60.7%)	26 (48.1%)	
Sarcomatoid differentiation			0.740
No	77 (91.7%)	51 (94.4%)	
Yes	7 (8.3%)	3 (5.6%)	
Postoperative complications			**0.001**
No	47 (56.0%)	45 (83.3%)	
Yes	37 (44.0%)	9 (16.7%)	

Bold values means statistically significant (p < 0.05).

**Figure 2:**
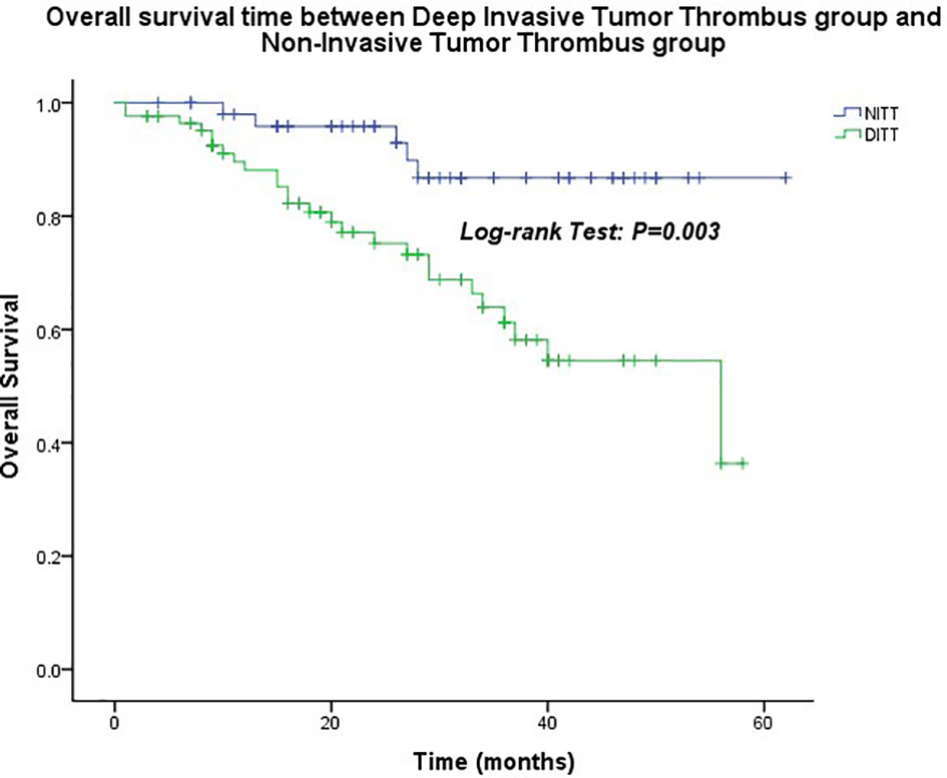
Overall survival time between the Deep Invasive Tumor Thrombus (DITT) group and the Non-Invasive Tumor Thrombus (NITT) group.

The median follow-up time was 26.0 months (1.0–62.0 months). The OS of the DITT group was less than that of the NITT group (41.5 ± 2.5 months vs. 56.7 ± 2.2 months), and the difference was statistically significant (*p* = 0.003) (**Figure 2**).

**Figure 3:**
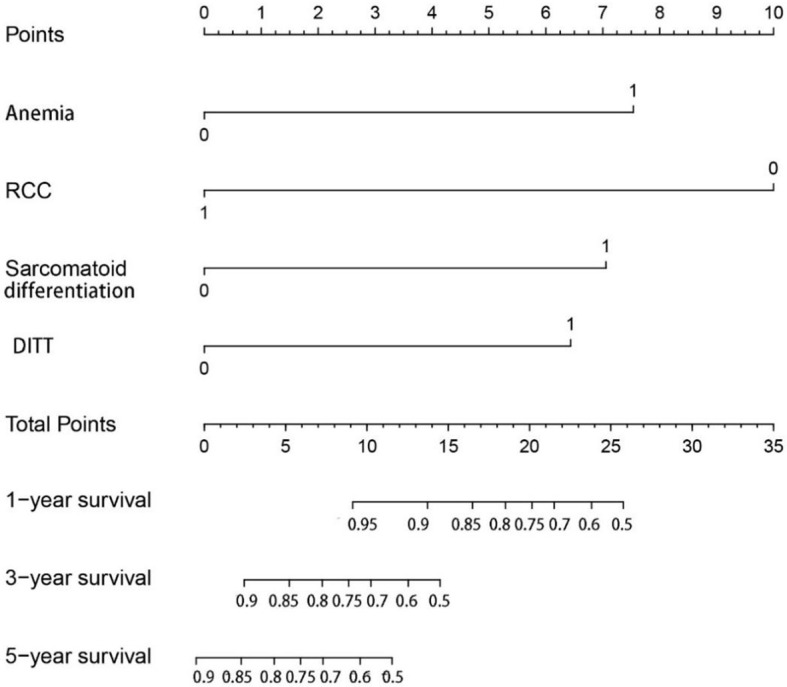
The nomogram can predict the 1-year, 3-year, and 5-year overall survival of patients with renal cell carcinoma with tumor thrombus.

In order to further study the impact of DITT on the prognosis, univariate and multivariate COX regression analyses were performed. The results are shown in **Table 2**. Multivariate analysis showed that sarcomatoid differentiation ([HR] = 2.968 [CI: 1.054–8.360], *p* = 0.040), DITT ([HR] = 2.781 [CI: 1.049–7.377], *p* = 0.040), low hemoglobin ([HR] = 3.159 [CI: 1.472–6.780], *p* = 0.003), and clear RCC ([HR] = 0.212 [CI: 0.091–0.496], *p* < 0.001) were independent prognostic risk factors of overall survival rate. Based on these four important variables, a nomogram for predicting 1-year, 3-year, and 5-year OS of non-metastatic RCC with VTT was constructed (**Figure 3**). By adding the scores associated with each variable and projecting the total scores to the bottom, OS probabilities can be estimated at 1-, 3-, and 5-year time points. For example, if a person has DITT, anemia, sarcomatoid differentiation, and clear cell carcinoma, his risk score is 21 (6.5 + 7.5 + 7 + 0) points, the predicted 1-year survival rate is approximately 70%, and the 3-year survival rate is less than 50%.

**Table 2:** Univariate and multivariate Cox regression analysis of renal cancer combined with tumor thrombus.

Item	Univariable analysis			Multivariable analysis		
	HR	95% CI	*p*-value	HR	95% CI	*p*-value
DITT	3.909	1.499–10.197	0.005	2.781	1.049–7.377	0.040
Hb	4.289	2.044–8.998	<0.001	3.159	1.472–6.780	0.003
Sarcomatoid differentiation	2.804	1.062–7.403	0.037	2.968	1.054–8.360	0.040
Clear RCC	0.171	0.076–0.381	<0.001	0.212	0.091–0.496	<0.001
IVC resection	2.910	1.389–6.095	0.005	—	—	0.712
Operative approach	1.729	0.826–3.618	0.146	—	—	0.944
Operative time	1.004	1.001–1.006	0.003	—	—	0.658
Surgical bleeding volume	1.000	1.000–1.000	0.050	—	—	0.408
Mayo classification	1.549	1.173–2.045	0.002	—	—	0.294
Elevated alkaline phosphatase	2.823	1.145–6.961	0.024	—	—	0.065
Fuhrman Grade	2.535	1.133–5.674	0.024	—	—	0.326
Clinical symptoms	2.278	1.374–3.778	0.001	—	—	0.290
Neu	1.071	0.935–1.226	0.322	—	—	0.544
Alb	0.922	0.874–0.973	0.003	—	—	0.051
Postoperative hospital stay	1.000	0.998–1.002	0.885	—	—	0.495

**Figure 4:**
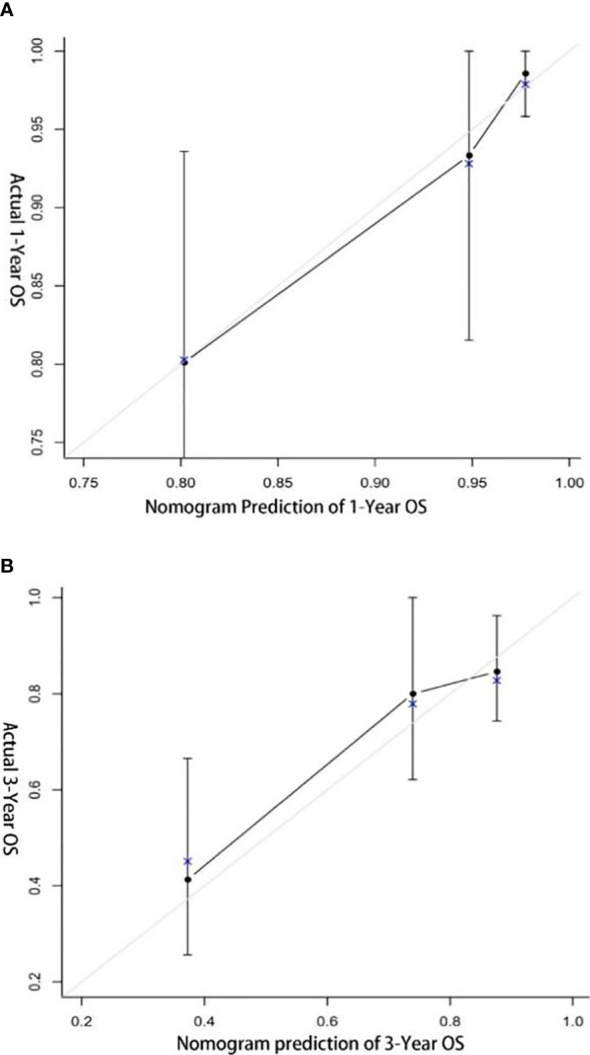
Calibration plot of nomogram. **(A)** 1-year overall survival; **(B)** 3-year overall survival.

The C-index of the nomogram for predicting OS of patients with non-metastatic RCC and VTT was 78.8% (CI: 71.2%–86.4%). The calibration plot of the nomogram is shown in **Figure 4**. The calibration plot showed good agreement of the nomogram between predicted and observed outcomes in 1- and 3-year survival predictions, which confirmed the reliability of the nomogram.

## Discussion

Preoperative prediction of whether the VTT grows adhesively or even invasively to the venous wall is very critical for optimal planning of the operation and rational evaluation of the patient’s prognosis. It is generally believed that enhanced MRI helps to diagnose the presence of DITT before surgery. The main predictive features include discontinuity of the vessel wall and complete occlusion of the IVC. It has high sensitivity (92%) and specificity (86%) (5). Some literature pointed out that the most vulnerable area of the IVC wall is the renal vein ostium. When the IVC diameter at the renal vein ostium is not less than 24.0 mm, the probability of DITT is greater (6). In addition, the largest coronary diameter of the IVC is also considered to be one of the main predictive features of DITT (7). In a study about contrast-enhanced ultrasound, the continuity of the IVC wall is considered to be a manifestation of DITT, and its prediction accuracy could reach 93.3% (8). However, it should be noted that various imaging examinations are highly dependent on the empirical judgment of the readers, and are limited by the consistency of the examination equipment, so the accuracy and repeatability are limited. In previous studies, the gold standard for diagnosis of DITT is generally intraoperative findings (5, 7, 8). Therefore, for IVC tumor thrombus, DITT in this study was also based on intraoperative findings. For RVTT, because the RV is directly cut off during the operation, the DITT cannot be observed in operation, so the DITT of the RVTT was confirmed by pathological examination. We also checked the CT images, and the accuracy of predicting the DITT was 82.1%. Since we have confirmed DITT at multiple levels, we believe that our research has a high level of reliability.

Radical nephrectomy and thrombectomy can effectively improve the prognosis of patients with RCC and VTT. The purpose of surgical treatment is to extensively remove the tumor burden. However, DITT significantly increases the difficulty of complete removal of VTT. Among our study, compared with the NITT group, Surgeries in the DITT group were much more difficult, which was reflected in higher possibility of transferring to open surgery, longer operation time, more blood loss, and more transfusion rate. Urological surgeries have contributed to the increasing prevalence of minimally invasive robot-assisted procedures (9). However, due to our own situation in which the Da Vinci system was just deployed within 1 year, we are currently collecting data of robotic surgeries as well. Therefore, we could not analyze the impact of DITT on robot-assisted surgery in this study and we are going to report it in the future. Correspondingly, the recovery of DITT group patients was more difficult, with longer hospital stay and more postoperative complications. Chung et al. found that racial factor is associated with a markedly elevated rate of major complications (10), but the population included in our study is all Chinese, avoiding the influence of racial factor. This is consistent with our experience and previous research (11). The reason might be as follows: (A) The proportion of high-level VTT was higher in the DITT group, with level III–IV accounting for 23.8%, while it was 9.3% in the NITT group. The surgical complexity of high-level VTT is higher than that of low-level VTT. (B) Venous wall invasion or invasion of DITT makes it difficult to peel off the tumor thrombus. Sometimes, segmental IVC resection or IVC ligation was necessary to achieve basic oncological control. In these cases, balloon catheter or milking technique was not appropriate to simplify the operative procedure. Previous studies have reported that about 6%–8% of patients with RCC combined with TT require segmental IVC resection (6). In our study, 29.5% of the DITT group performed segmental resection of IVC. (C) DITT represents a certain subtype of tumor with highly aggressiveness and exudative inflammation around paratumoral matrix, which requires more time for separation.

The impact of DITT on the prognosis is still controversial. A systematic review showed that large tumor size, high Fuhrman grade, tumor necrosis, positive lymph node, and metastasis at surgery were significant adverse predictors for both CSS and OS. Also, IVC tumor thrombus, sarcomatoid differentiation, perirenal fat infiltration, and adrenal gland invasion were associated with poor CSS (12). However, the effect of DITT on the prognosis was not found. However, some studies hold the opposite opinion that DITT can lead to worse prognosis of RCC patients (13). DITT can provide a suitable matrix for the invasion of bland thrombus, thereby facilitating the formation of bland thrombus. Bland thrombus is related to poor prognosis of RCC with VTT (14). In order to accurately evaluate the impact of DITT on the prognosis, we excluded patients in T4, N1, and M1 status. The results showed that the OS of DITT patients was shorter than that of NITT patients (*p* = 0.032). As for the reasons, on the one hand, DITT might indicate that the tumor is more aggressive and has worse biological behavior. On the other hand, invasion of the venous wall corresponds to higher surgical difficulty and perhaps compromised oncologic control.

In the multivariate analysis of the current study, only anemia, sarcomatoid differentiation, non-clear cell carcinoma, and DITT were associated with poor prognosis. Anemia (low hemoglobin) represents a long-term poor diet or high consumption of the tumor. In previous studies, blood indicators such as hemoglobin and neutrophils have been reported to be related to the prognosis (15, 16). Sarcomatoid differentiation is currently considered a rare histologic variant that predicts aggressive behavior and poor prognosis (17). RCC with sarcomatoid differentiation is classified as grade 4 by the WHO/ISUP grading system (18). Even after adjusting for multiple other characteristics, sarcomatoid differentiation still leads to a 3.2 times higher risk of CSS (19). Different histological types have different clinicopathological characteristics and prognosis. Ciancio et al. (20) found that higher nuclear grade, distant metastasis, and non-clear RCC were independent prognostic factors for poor survival. A large sample study also showed that compared with clear RCC, papillary RCC has a worse prognosis (21). Based on these four factors, we made a nomogram to predict the OS of T3N0M0 RCC with VTT. The nomogram demonstrated satisfied predictive value with a C-index of 78.8% (CI: 71.2%–86.4%). Very perfect agreement was observed in the calibration plot of our nomogram between the predicted and observed outcomes in 1- and 3-year survival predictions. Therefore, our prognostic nomogram may aid clinicians in predicting the survival outcome of nmRCC with VTT patients and provide the reference for therapy guidance.

This study has some limitations. First, this study is a retrospective study with a single-center experience, and there is some selection bias. Secondly, for IVCTT, the DITT was an intraoperative diagnosis and not diagnosed at a preoperative imaging (with the possibility of a not negligible false positive rate). The judgment found during the operation is subjective and is not entirely the exact invasion of the vein wall. The invasion could be caused by inflammation around the tumor, so there is a certain false-positive rate, which is one of the research limitations of our research. Finally, the outcome indicator of this study is overall survival, and there is a lack of information on progression-free survival. In the follow-up, further large-sample prospective studies are needed to provide more evidence.

According to the results of this study, DITT can significantly increase the difficulty of surgery for patients of RCC with VTT, and may lead to poor prognosis. Multivariate analysis showed that sarcomatoid differentiation, DITT, low hemoglobin, and pathological type were independent prognostic risk factors. We constructed a valuable prediction nomogram to predict prognosis of nmRCC with VTT well, which might provide a reliable prognosis assessment tool for clinician and aid treatment decision-making in the clinic.

## Data Availability Statement

The raw data supporting the conclusions of this article will be made available by the authors, without undue reservation.

## Ethics Statement

The studies involving human participants were reviewed and approved by the Peking University Third Hospital ethics committee. The patients/participants provided their written informed consent to participate in this study. Written informed consent was obtained from the individual(s) for the publication of any potentially identifiable images or data included in this article.

## Author Contributions

XZ and YY: study conception and design, literature search, clinical studies, data analysis, statistical analysis, manuscript preparation, and manuscript editing. J-HD, ZL, and H-XZ: study conception and design, literature search, clinical studies, data analysis, and manuscript editing. CL and L-LM: guarantor of the integrity of the entire study. The authors have read and approved this manuscript, and ensure that the listed authors’ contributions are accurate.

## Funding

This study was supported by the National Nature Science Foundation of China (No. 81771842).

## Conflict of Interest

The authors declare that the research was conducted in the absence of any commercial or financial relationships that could be construed as a potential conflict of interest.

## Publisher’s Note

All claims expressed in this article are solely those of the authors and do not necessarily represent those of their affiliated organizations, or those of the publisher, the editors and the reviewers. Any product that may be evaluated in this article, or claim that may be made by its manufacturer, is not guaranteed or endorsed by the publisher.

## Abbreviations

RCC, renal cell carcinoma; VTT, venous tumor thrombus; OS, overall survival; IVC, inferior vena cava; ASA, American Society of Anesthesiologists; HR, Hazard ratios; BMI, body mass index; DITT, deep invasive tumor thrombus; NITT, non-invasive tumor thrombus.
